# Perfectionism Mediates the Relationship Between Parental Expectations and Adolescent Depressive Symptoms

**DOI:** 10.3390/bs16010125

**Published:** 2026-01-15

**Authors:** Tolulope S. Aworefa, Kathryn L. Fletcher

**Affiliations:** Department of Educational Psychology, Ball State University, Muncie, IN 47306, USA; klfletcher@bsu.edu

**Keywords:** perfectionism, parental expectations, depression, social expectations, adolescents, Global South

## Abstract

A significant body of research worldwide has examined how parents who set high expectations may increase adolescents’ risk of developing perfectionistic traits. However, studies exploring this relationship in the Global South are almost nonexistent. This study investigated how adolescents perceived parental expectations related to perfectionism and depressive symptoms among Nigerian adolescents. Participants completed the Frost Multidimensional Perfectionism Scale (FMPS), the Living Up to Parental Expectation Scale—Academic (LPE), and Beck’s Depression Inventory. Parental academic expectations were positively associated with personal standards and concern over mistakes, but parental expectations were negatively associated with depressive symptoms. Further analysis revealed that personal standards fully mediated the negative relationship between parental academic expectations on adolescents’ depressive symptoms. In contrast, concern over mistakes partially mediated the relationship between parental academic expectations and depressive symptoms. In contrast to previous research, parental academic expectations were associated with fewer depressive symptoms among Nigerian adolescents through indirect relationships with perfectionistic traits.

## 1. Introduction

Parent expectations for adolescents often serve to guide their goals, performance, and behaviors during a critical time period when they are struggling with their academic identity. When parental expectations for adolescents’ academic performance match the adolescents’ goals and are realistic, adolescents benefit from high parental expectations in their academic work (Patterson et al., 2021). In other words, adolescents who see high parental expectations as messages that they are capable and can succeed are likely to feel supported by their parents. Realistic parental expectations can motivate adolescents and boost their persistence toward their goals (Wigfield & Eccles, 2000). Conversely, adolescents might interpret these expectations as unrealistic or mismatched, surpassing their own capabilities, which can lead to lower academic achievement (Cross et al., 2019). Besides harming adolescents’ academic success, parents’ unrealistic and excessive demands increase psychological distress in adolescents (Ma et al., 2018; Lu et al., 2020).

Empirical evidence has supported the notion that parental expectations are an antecedent of the development of perfectionistic traits (Madjar et al., 2015; Stoeber & Otto, 2006). Perfectionistic traits involve two dimensions: exceedingly high standards for one’s performance (i.e., perfectionistic strivings), and/or extreme self-criticism and hypersensitivity to less-than-perfect performance (i.e., perfectionistic concerns). According to the social expectation model, adolescents’ internalization of expectations from significant others shapes their behavior, self-evaluations, and motivation (Flett & Hewitt, 2022). When parental expectations are harshly persistent and unattainable, adolescents internalize these demands as perfectionistic traits and strive to meet the expectations to gain parental love, acceptance, and emotional closure (Flett & Hewitt, 2022). Moreover, despite achieving objectively successful outcomes, individuals with perfectionistic traits can become trapped in extreme self-criticism and dissatisfaction, which reduces their life satisfaction and negatively impacts their mental health (Stoeber & Otto, 2006). Perfectionism has consistently been linked to rising rates of adverse psychological outcomes such as anxiety, depression, and self-criticism over the last several decades (Curran & Hill, 2022).

Although parental expectations have been related to perfectionism and rising rates of negative mental health outcomes, studies have been disproportionately focused on Western, educated, industrialized, rich, and democratic (WEIRD) populations. Research on perfectionism and its consequences must be expanded to include diverse cultural contexts (Smith et al., 2022). The ecological niche of the Nigerian environment, characterized by normalized academic competition, cultural values that prioritize respect for elders, and limited mental health services, warrants further study. There is a paucity of research that has investigated the influence of culturally salient parental academic expectations on perfectionism and adolescents’ mental health outcomes. The current research fills this gap by examining the association between parental academic expectations, perfectionism, and depressive symptoms in a sample of Nigerian adolescents.

### 1.1. Perfectionism and Parental Expectations

Perfectionism is a multidimensional personality trait comprising two dimensions: perfectionistic strivings and perfectionistic concerns (Stoeber & Otto, 2006). Perfectionistic strivings are when individuals set very high standards, are highly organized, feel satisfied when they meet their goals, and seek to improve without fear of failure (Slaney et al., 2001; Stoeber & Otto, 2006). In contrast, perfectionistic concerns are characterized by behaviors and attitudes such as worrying excessively about making mistakes, feeling distressed when they fall short of their own expectations, and striving to meet others’ high expectations (Frost et al., 1990; Hewitt & Flett, 1991; Limburg et al., 2017).

Over the decades, scholars have developed measures to assess individuals’ perfectionistic traits, with the most used measures containing items for both perfectionistic strivings and perfectionistic concerns. The Frost Multidimensional Perfectionism Scale (FMPS; Frost et al., 1990) includes six dimensions: concern over mistakes, doubt about actions, parental criticism, parental expectations, personal standards, and organization. However, most researchers only examine three of the measures (personal standard, concern about mistakes, and doubts about actions) following the recommendations outlined by Stoeber and Otto (2006). The Hewitt–Flett Multidimensional Perfectionism Scale (MPS; Hewitt & Flett, 1991) conceptualizes three perfectionism dimensions: self-oriented perfectionism (i.e., imposing standards for perfection on oneself), socially prescribed perfectionism (i.e., belief that significant others expect perfection), and other-oriented perfectionism (i.e., belief that others should be perfect). The Almost Perfect Scale-Revised (APS-R; Slaney et al., 2001) measures perfectionistic traits by indexing high standards, order, and discrepancy (i.e., congruence between standards and performance). Despite the various measures of perfectionism, the two-dimensional framework—perfectionistic strivings and perfectionistic concerns—as a way to discuss trends in perfectionism and its consequences has become the norm in perfectionism research (Stoeber & Otto, 2006). In the current study, perfectionistic strivings and perfectionistic concerns are used to describe trends in the literature; however, specific dimensions from different measures are used to describe prior research findings.

Adolescents’ exhibition of perfectionistic traits is influenced by various factors, including parental expectations, parental criticism, societal pressure, and early childhood experiences (Flett & Hewitt, 2022). Among these contributing factors, parental expectations and parental criticism have been documented as antecedents of perfectionism (Curran & Hill, 2022; Damian et al., 2021). In a longitudinal study, Domocus and Damian (2018) found that perceived parental expectations significantly predicted perfectionistic concerns in 14- to 19-year-olds from two high schools in Romania. Over a short period, parental expectations increased adolescents’ concern over mistakes and doubts about their actions. Consistent with this pattern, Menon et al. (2025) reported significant positive relationships among perceived parental expectations, perfectionistic concerns, and fear of negative evaluation. Using the Frost Multidimensional Perfectionism Scale-Brief, evaluative concerns (i.e., perfectionistic concerns) fully accounted for the relationship between parental expectations and fear of negative evaluation (Menon et al., 2025). Thus, adolescents whose parents have high expectations are at risk of developing perfectionistic concerns.

Adolescents’ perceptions of parental expectations are not always linked to perfectionistic concerns. When communicated in an autonomy-promoting environment, parental expectations can be connected to perfectionistic strivings (Damian et al., 2021). Lavrijsen et al. (2021) examined perfectionistic concerns and perfectionistic strivings, as well as parental influences, among adolescents with varying levels of cognitive ability. Sampling over three thousand Belgian adolescents, high parental expectations were positively related to adolescents’ personal standards. Similarly, using the FMPS sub-scale of personal standard and organization to measure perfectionistic strivings in Israeli adolescents, Madjar et al. (2015) reported that perceived high parental expectations fostered perfectionistic strivings among Israeli high school students.

More broadly, parental expectations can promote motivation and performance in adolescents when communicated properly within a supportive environment and perhaps a specific cultural context (Almroth et al., 2020; Nafiah & Izzaty, 2024). In contrast, parental expectations can also breed a fear of failure, negative evaluation, and critical self-judgment when internalized as unrealistic expectations (Menon et al., 2025). Therefore, parental expectations and their impact on perfectionism are shaped by interactions within family contexts, which can vary across cultural contexts.

### 1.2. Social Expectations Model

Theoretical models have also hypothesized that the family and cultural context may contribute to the development of perfectionism (Flett & Hewitt, 2022). Specifically, the social expectation model suggests that adolescents internalize the expectations of significant others, and perfectionism may arise when adolescents perceive their parents’ approval, love, and acceptance as tied to fulfilling those expectations (Flett et al., 2002; Flett & Hewitt, 2022). Adolescents may view parental expectations as the standard for their self-worth, which in turn influences their behavior. Meta-analysis findings by Curran and Hill (2022) indicated that adolescents’ perceptions of parental expectations and criticism have increased over the past several decades. Adolescents are often more concerned about meeting their parents’ expectations, which can put pressure on them. This pressure may originate from striving to meet the expectations of their parents, resulting in mental health outcomes such as depression, anxiety, and stress.

Haspolat and Yalçın (2023) examined how parental achievement pressure affected perfectionism and psychological outcomes among Turkish adolescents. Parental achievement pressures significantly predicted self-oriented perfectionism in adolescents; however, self-oriented perfectionism did not predict psychological distress. Supporting these results, Damian et al. (2013) explored the impact of parental expectations and parental criticism on perfectionism among Romanian adolescents. According to Damian et al. (2013), only parental expectations predicted long-term increases in socially prescribed perfectionism among adolescents. Conversely, parental expectations were not associated with self-oriented perfectionism.

To further support this theory, adolescents who internalize parental expectations and view them as unachievable and unrealistic tend to focus more on their mistakes, failures, and flaws. Examining high school students in Israel, Madjar et al. (2015) found that parental expectations predicted personal standards and concern about mistakes, but the relationship was stronger for personal standards. However, parental criticism predicted adolescents’ concern over mistakes. Overall, internalization of expectations as unachievable increases an individual’s perceived frequency of mistakes, flaws, and harsh self-judgment.

The social expectations model has provided an explanation of how perfectionistic behavior and psychological distress may occur through parental expectations. For example, when adolescents sense that parental expectations are conditional, unattainable, or contingent, internalization can lead to perfectionistic concerns. On the other hand, the internalization can also lead to self-supported standards and striving for excellence when perceived expectations are realistic, supportive, and culturally normative. Therefore, parents’ expectations for their adolescents must be examined within the cultural context. To our knowledge, there has been limited research that has examined adolescents’ perfectionism and its consequences for mental health within the cultural context of different African countries. Within different cultural contexts, parental behaviors may be viewed as the “norm” for a population and thus may impact the extent to which adolescents internalize their parents’ expectations. Differences in how adolescents internalize parental expectations may influence the development of perfectionistic traits and their consequences.

### 1.3. Perfectionism and Mental Health Outcomes

Self-criticism is one of the hallmarks of clinical depression. However, high levels of self-criticism can place individuals at risk for suffering from subclinical depressive symptoms. As perfectionistic concerns increase self-criticism, individuals with high levels of perfectionistic concerns are at risk for depression (Limburg et al., 2017; Lunn et al., 2023). In a longitudinal study, Levine et al. (2019) reported that adolescents’ self-critical perfectionism (i.e., concern over mistakes from the FMPS) increased depressive symptoms across the school year. Using the Almost Perfect Scale, Doyle and Catling (2022) demonstrated that discrepancy, a form of perfectionistic concerns characterized by a discrepancy between one’s performance and one’s standards, predicted both depression and anxiety in young people.

Socially prescribed perfectionism, the belief that others hold high expectations, has been investigated as a direct predictor of poor mental health outcomes. Flett et al. (2016) reported strong relationships between socially prescribed perfectionism and mental health outcomes such as high depressive symptoms, greater stress, low self-esteem, and maladaptive attitudes towards eating. A recent systematic review supported these findings: socially prescribed perfectionism had a strong relationship with adverse mental health outcomes such as depression, suicidal ideation, and self-harm (Patterson et al., 2021).

Building on previous findings, Molnar et al. (2022) conducted a three-wave longitudinal study of Canadian adolescents to examine how perfectionistic traits relate to anxiety, depression, and stress before and during the COVID-19 pandemic. Using multilevel growth models, socially prescribed perfectionism was a strong predictor of psychological distress. Higher socially prescribed perfectionism consistently predicted increases in depressive symptoms; however, self-oriented perfectionism was not consistently linked to depression once other factors, such as stress, were controlled. The difference highlights that adolescents who perceived pressure from others to be perfect are vulnerable to experiencing depression.

Most empirical studies exploring the links between perfectionism dimensions and mental health outcomes have been conducted in contexts outside the global south. Consequently, there is a substantial gap in understanding the dynamics of perfectionistic dimensions shaping adolescents’ mental health within the Global South. To address this limitation, the present study investigated the influence of parental academic expectations on perfectionism and mental health in Nigerian adolescents.

### 1.4. Current Study

The current research examined the association between parental academic expectations, multidimensional perfectionism, and depressive symptoms in a sample of Nigerian adolescents. Perfectionism has been widely studied in Western contexts, yet little is known about how parental expectations shape perfectionistic traits and mental health outcomes among adolescents in non-WEIRD societies. Nigeria, like other Global South countries, places a strong cultural value on high academic achievement within competitive environments, as well as respect for elders such as parents. Therefore, this study explored how parental academic expectations influence the perfectionistic traits and their consequences on adolescents’ mental health. Grounded in the social expectation theory (Flett & Hewitt, 2022), we propose that: (1) parental academic expectations would be associated with perfectionism dimensions (personal standards and concern over mistakes), and (2) perfectionism dimensions would predict depressive symptoms.

## 2. Materials and Methods

### 2.1. Participants

The study sampled 285 secondary school students (M = 16.64) comprising 119 males and 166 females aged 12–18. Participants were selected from three private Senior Secondary Schools (S.S.S.) in the city of Ile-Ife, Osun State, within the senior classes 1–3 range to ensure adequate class sizes. The school selected ran two schooling systems (day and boarding schools). The sample reflects private secondary school students attending private schools in Ile-Ife. The school principals, on behalf of the Parents and Teachers Associations (PTA), granted permission and consent for the study to be conducted. Students who agreed to participate in the study provided consent, and their participation was anonymous. The study questionnaires were administered to the participants at regular classroom time under the guidance of the first author, a trained research assistant, and a school counselor, who were also present to explain the instructions and answer the questions.

### 2.2. Measures

#### 2.2.1. Living up to Parent Expectations Inventory

The Living Up to Parent Expectations inventory (Wang & Heppner, 2002) assessed the participants’ perceived parental expectations. Although Wang and Heppner’s (2002) inventory includes multiple domains, we only used the academic achievement sub-scale to measure the adolescents’ perceived parental expectations. Responding to each item, participants were prompted to respond to how they perceived parents’ expectations regarding their academics on a 5-point Likert-type scale (1 = not at all expected; 2 = somewhat expected; 3 = expected; 4 = strongly expected, 5 = very strongly expected). The Cronbach alpha reliability coefficient for this measure was 0.78. For data analysis, we refer to this variable as “parental academic expectations”.

#### 2.2.2. Frost’s Multi-Dimensional Perfectionism Scale

The Frost Multidimensional Perfectionism Scale (Frost et al., 1990) was used to measure perfectionism on six sub-scales (organization, parental expectations, parental criticism, doubt about actions, concern over mistakes, and personal standards). However, following the methods of other researchers on perfectionism, we focused only on concerns about mistakes and personal standards (Gäde et al., 2017; Mallinson & Hill, 2011; Smith et al., 2019). Although it was initially developed for adults, the Frost Multidimensional Perfectionism Scale has been used in adolescent samples in prior research (Gyori et al., 2023; Sironic & Reeve, 2015). Each item was rated on a 5-point Likert-type scale (1 = strongly disagree; 2 = disagree; 3 = neither disagree nor agree; 4 = agree; 5 = strongly agree). Internal consistency, as measured by Cronbach’s alpha, was 0.71 for personal standards and 0.70 for concern over mistakes.

#### 2.2.3. Beck Depression Inventory

The Beck Depression Inventory (Beck et al., 1988) was used to measure the presence and severity of depressive symptoms in the past 7 days. The Beck Depression Inventory has also been used with adolescent populations in both clinical and non-clinical research due to its strong psychometric properties and coverage of depressive symptoms (Lee & Park, 2022). It comprised 21 items that measure symptoms such as sadness, pessimism, past failure, and guilt, among others. Two sensitive items, loss of interest in sex and suicidal thoughts, were excluded as being culturally irrelevant for a Nigerian adolescent population. Each item was scored from 0 to 3 based on intensity, and the participants were asked to circle the options that best described their feelings (0 = absence of symptom to 3 = severe symptom). The internal consistency using Cronbach’s alpha was 0.83.

### 2.3. Data Analysis

The researchers used SPSS version 28 for data analysis. During data entry, it was determined that the sample had no missing cases. Descriptive statistics for the data, the mean and standard deviation, were obtained (see Table 1). Two variables, personal standards and living up to parental expectations, were found to be moderately negatively and positively skewed, respectively. A square-root transformation was used for the positively skewed variable, while the negatively skewed variable was first reflected before applying the logarithmic transformation. Although gender differences were not a major variable for our research questions, preliminary analyses examined gender differences across the study variables. No statistically significant gender differences were found for personal standards, concern over mistakes, or depressive symptoms (*t*s < 0.83, *p*s > 0.05). A gender difference was found for parental academic expectations (*t* (283) = −0.74; *p* < 0.05), but the effect size was negligible (Cohen’s d = −0.09). Because gender was not meaningfully associated with the primary variables, it was not included in the primary analytic models. The correlation and regression analysis in this study was carried out using the transformed scores. Notably, the substantive interpretation of the findings was the same whether transformed or untransformed scores were used. Other assumptions, such as independence, multicollinearity, homoscedasticity, and linearity, were not violated. A bivariate Pearson’s correlation was first conducted to examine the relationships among study variables. Mediation analysis was subsequently performed using Hayes’ PROCESS macro (version 4.0), employing Model 4 with 5000 bootstrap samples to estimate the indirect effects.

## 3. Results

Descriptive statistics and correlations are presented in Table 1. Depression was negatively associated with both personal standards (r = −0.24, *p* < 0.01) and parental academic expectation (r = −0.14, *p* < 0.05) but positively associated with concern over mistakes (r = 0.25, *p* < 0.01). Furthermore, parental academic expectations showed a positive significant relationship with personal standards (r = 0.44, *p* < 0.01) and concerns over mistakes (r = 0.23, *p* < 0.01).

The regression analysis (Table 2) revealed a model accounting for 6% of the variability in depressive symptoms (R^2^ = 0.06, F(2, 282) = 8.98, *p* < 0.001). Personal standards were the only robust predictor of depressive symptoms, showing a significant negative association (*β* = −0.23, *p* = <0.001), while parental academic expectations did not significantly predict depressive symptoms in the direct model (*β* = −0.04, *p* = 0.58). A significant indirect effect (ab = −0.18, 95% CI [−0.29, −0.08]) indicated that personal standards fully mediated the association between parental academic expectations and depressive symptoms.

**Figure 1:**
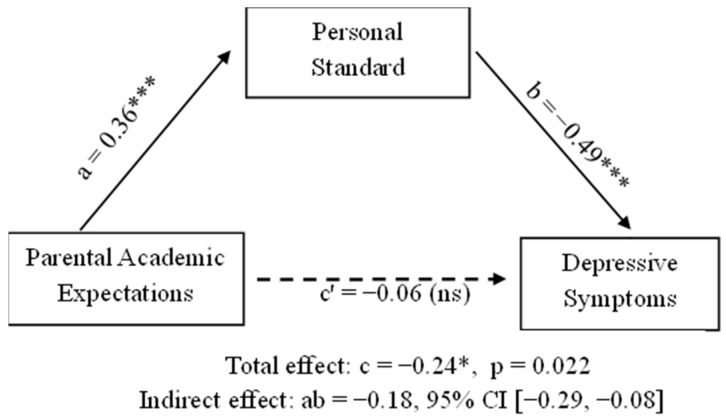
Direct and indirect effects for the mediation model examining the relationship between parental academic expectations and depressive symptoms, mediated by personal standards. *** *p* < 0.001; * *p* < 0.05; ns—not significant.

The mediation model tested whether personal standards explained the link between parental academic expectations and depressive symptoms. As shown in Figure 1, higher parental expectations significantly predicted higher personal standards (path a = 0.36, 95% CI [0.28, 0.45], *p* < 0.001), and personal standards were associated with lower depressive symptoms (path b = −0.49, 95% CI [−0.77, −0.22], *p* < 0.001). The direct effect of parental expectations on depressive symptoms was not significant when personal standards were included (path c′ = −0.06, 95% CI [−0.29, 0.16], *p* = 0.58), although the total effect remained significant (path c = −0.24, 95% CI [−0.45, −0.04], *p* = 0.022). The indirect effect was significant (ab = −0.18, 95% CI [−0.29, −0.08]), indicating that personal standards fully mediated the relationship.

Bootstrapped confidence intervals supported a significant indirect effect of parental academic expectations on depressive symptoms through personal standards, ab = −0.18, BootSE = 0.05, 95% CI [−0.29, −0.08], indicating full mediation. These findings suggest that parental expectations are statistically associated with depressive symptoms indirectly through personal standards, such that the direct association between parental academic expectations and depressive symptoms is no longer evident once personal standards are included in the model.

The regression analysis (Table 3) revealed a model accounting for 10% of the variability in depressive symptoms (R^2^ = 0.10, F(2, 282) = 16.47, *p* < 0.001). Concern over mistakes was the most robust predictor of higher depressive symptoms (*β* = 0.30, *p* < 0.001), followed by parental academic expectations in a negative direction (*β* = −0.21, *p* < 0.001). A significant indirect effect (ab = 0.13, 95% CI [0.05, 0.22]) indicated that concern over mistakes partially mediated the association between perceived parental academic expectation and depressive symptoms.

**Figure 2:**
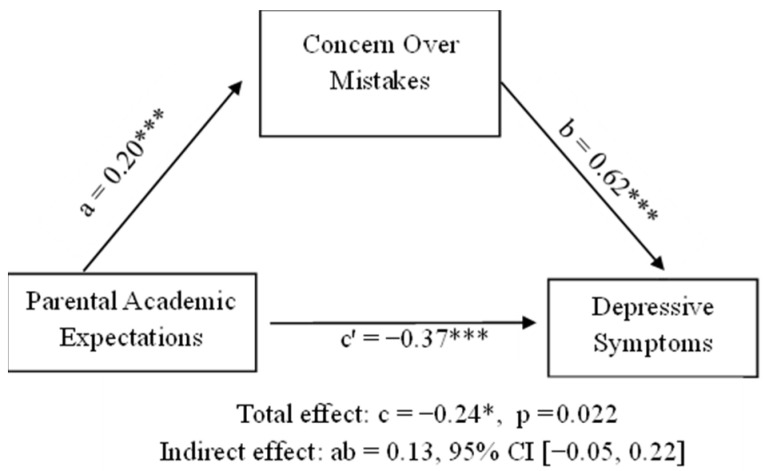
Direct and indirect effects for the mediation model examining the relationship between parental academic expectations and depressive symptoms, mediated by concern over mistakes. *** *p* < 0.001; * *p* < 0.05.

The mediation model tested whether concern over mistakes mediated the association between parental academic expectations and depressive symptoms. As illustrated in Figure 2, higher parental expectations significantly predicted greater concern over mistakes (path a = 0.20, 95% CI [0.10, 0.30], *p* < 0.001), and concern over mistakes was strongly associated with higher depressive symptoms (path b = 0.62, 95% CI [0.38, 0.85], *p* < 0.001). The direct effect of parental expectations on depressive symptoms remained significant when concern over mistakes was included (path c′ = −0.37, 95% CI [−0.57, −0.16], *p* < 0.001), while the total effect was smaller (path c = −0.24, 95% CI [−0.45, −0.04], *p* = 0.022). A significant indirect effect (ab = 0.13, 95% CI [0.05, 0.22]) indicated that concern over mistakes partially mediated this relationship.

Bootstrapped confidence intervals supported a significant indirect effect of parental academic expectations on depressive symptoms through concern over mistakes, ab = 0.13, BootSE = 0.04, 95% CI [0.05, 0.22], indicating partial mediation. These findings suggest that parental expectations associated with depressive symptoms are both directly and indirectly related to concern over mistakes.

## 4. Discussion

The current study investigated the relationship between parental academic expectations, perfectionism traits (personal standards and concern over mistakes), and depressive symptoms among Nigerian adolescents. Nigerian adolescents’ perceptions of their parents’ academic expectations were positively associated with personal standards and concern over mistakes. These findings aligned with prior work linking parental expectations to adolescents’ perfectionistic strivings and perfectionistic concerns in Western countries (Curran & Hill, 2022; Smith et al., 2022; Stoeber & Rambow, 2007). Consistent with the social expectations model, parental expectations, even in non-Western cultures, may be an antecedent of adolescents’ perfectionistic traits (Flett & Hewitt, 2022). Adolescents who perceive parental expectations as realistic, who are supported in their abilities by their parents, and who feel encouraged to succeed may tend to internalize these expectations as personal standards. On the other hand, unrealistic and overly demanding parental expectations may lead to perfectionistic concerns (concern over mistakes), characterized by worries, fear of failure, and anxiety about disappointing their parents. Such adolescents become concerned about their capacity to meet expectations and may criticize themselves if they fall short. In this context, adolescents may exhibit perfectionistic concerns to gain approval and validation from their parents (Flett & Hewitt, 2022).

Inconsistent with prior research, however, Nigerian adolescents perceived parental academic expectations were negatively and significantly related to depressive symptoms, indicating that adolescents who perceived that their parents had high academic expectations tended to report lower depressive symptoms. A potential reason for the negative association between parental academic expectations and depressive symptoms is the interpretation and internalization of these expectations within the Nigerian sociocultural framework. In Nigerian environments where parental authority is highly valued, interdependence is strong, and education is highly valued as a pathway to future stability, academic expectations may provide a structure, guidance, and a perceived investment in the adolescent’s future rather than exerting pressure. In Nigerian culture, parents believe that parental academic expectations equip adolescents with purpose and clarity about their future, which serves as protection against depressive symptoms. Previous research has reported that parents’ educational expectations can positively impact adolescents’ academic pursuits and reduce depressive symptoms (Liu et al., 2022). Conversely, many other studies have found significant relationships between adolescents’ reports of depression and parental expectations (Díez et al., 2024; Haspolat & Yalçın, 2023; Okereke & Ebenuwa-Okoh, 2021; Stiles et al., 2020). Discrepancies in these results might be explained from a cultural perspective. In a collectivist society where adolescents respect their elders’ wishes, parental expectations may be internalized differently compared to an individualist society (Menon et al., 2025; Sawitri et al., 2015).

As support for the connection between parental expectations and depression in Nigeria, Okereke and Ebenuwa-Okoh (2021) reported that parental expectations were positively related to undergraduate students’ depression. However, when financial stress was included in the regression model, the relationship between parental expectations and depression became non-significant. The development stage and situational needs could explain this difference. Adolescents in secondary school, as in the current study, are generally shielded from financial responsibility, as all financial obligations rest solely on their parents. Therefore, adolescents perceived parental academic demands as preparatory guidance and encouragement. Conversely, undergraduates encounter greater academic, economic, and employment demands, which can turn parental expectations into stressors, especially when financial strain is salient. To put it simply, parental expectations are viewed as encouraging or supportive during adolescence, yet overwhelming when linked to increased demands for autonomy and financial pressures in emerging adults.

Despite our findings that parental expectations negatively predicted depressive symptoms, perfectionistic concerns and perfectionistic standards played a role in this relationship. Personal standards were a significant and negative predictor of depressive symptoms, and personal standards fully mediated the relationship between parental academic expectations and depressive symptoms. This finding is supported by previous studies that reported that adolescents with perfectionistic striving traits experienced fewer depressive symptoms than those with perfectionistic concern traits (Kuusi et al., 2025; Levine et al., 2019; Lunn et al., 2023; Smith et al., 2021). The small amount of variance accounted for suggests that parental academic expectations and perfectionistic traits are related to depressive symptoms but constitute only a subset of the contributing factors. A broader set of factors, including financial stress, peer relationships, school climate, and adverse life events, likely contributed to the adolescents’ depressive symptoms. Moreover, more general parenting styles should be investigated in relation to parental academic expectations, as how messages about academic expectations might be delivered in consistently harsh and insulting ways (e.g., authoritarian parenting) or supporting and affirming ways (e.g., authoritative parenting) that may impact adolescents’ depressive symptoms. When adolescents’ perfectionism evolves from their own internal drive and motivation, perhaps as parental academic expectations have become more internalized, it may be associated with lower depressive symptoms in Nigerian adolescents.

However, concern over mistakes positively predicted depressive symptoms, consistent with prior studies (Flett et al., 2016; Patterson et al., 2021) and two meta-analyses (Lunn et al., 2023; Smith et al., 2021). When examining unique variance related to concern over mistakes, parental academic expectations continued to serve as a significant negative predictor of depressive symptoms, indicating only partial mediation. Adolescents who are overly worried about making mistakes are more vulnerable to experiencing emotional distress, even though parental expectations might help reduce depressive symptoms. Whereas other previous research on perfectionistic concerns, parenting, and depression has reported positive associations among variables (Stiles et al., 2020), this was not the case in our sample of Nigerian adolescents. As indicated in a review of the state of research on perfectionism after 30 years, research on non-WEIRD samples and cross-cultural research is needed to better understand the consequences of perfectionism across different cultural contexts (Smith et al., 2022).

### Limitations

To our knowledge, this study represents the only research on perfectionism and depressive symptoms in an African country. A limitation of this study is that it only focused on Nigerian adolescents. Parenting styles, societal expectations, and cultural norms in Nigeria may differ from those in other African countries, potentially limiting the applicability of the findings to other African countries. We also relied on adolescents’ self-reports about their perceptions of their parents’ academic expectations. Research has shown that the “perception” of parenting behaviors can be more influential in predicting outcomes than parental reports of parenting behaviors (Choe, 2020; De Los Reyes et al., 2019). Additionally, in our sample of adolescents, perfectionistic traits were assessed using a self-report tool designed explicitly for emerging adults and adults. Therefore, our measure may not have fully captured the developmental nuances of perfectionism in adolescents. Although Cronbach’s alpha indicated that the items for each perfectionism subscale were acceptable, future research should employ a measure of perfectionism designed for adolescents, such as the Child and Adolescent Perfectionism Scale (CAPS; Flett et al., 2016). Due to the cross-sectional nature of this study, causality cannot be inferred, nor can the directionality of the effects between parental expectations, perfectionism dimensions, and depressive symptoms be determined. Therefore, future studies should incorporate additional theoretically relevant variables to strengthen causal interpretation. Lastly, although we found associations between parental academic expectations and perfectionism, one important area for future research will be to examine adolescents’ actual academic performance and how their performance is aligned with their parents’ academic expectations. Adolescents’ failure to live up to parental academic expectations might have a stronger association with depressive symptoms related to perfectionistic concerns.

## 5. Conclusions

This study explored the relationship between perceived parental academic expectations, perfectionism dimensions, and depressive symptoms among Nigerian adolescents, addressing a significant gap in the non-Western context. Overall, perceived parental expectations were positively associated with personal standards and concern about mistakes, but negatively associated with depressive symptoms. Further analysis clarified this relationship through the mediating role of perfectionism dimensions. These findings suggest that perceived parental academic expectations generally correlated with fewer depressive symptoms among these Nigerian adolescents. The relationship between lower depressive symptoms was most evident when adolescents internalized parental expectations as their own standards. Nevertheless, adolescents who expressed concern over mistakes were more susceptible to depressive symptoms.

## Figures and Tables

**Table 1:** Descriptive Statistics and Correlation Matrix Among Study Variables.

	1	2	3	4
Depressive Symptoms	1			
Personal Standard	−0.24 **	1		
Concern over Mistakes	0.25 **	0.13 *	1	
Parental Academic Expectation	−0.14 *	0.44 **	0.23 **	1
Mean	2.57	3.81	2.96	3.50
Standard Deviation	1.46	0.67	0.71	0.82
Alphas (α)	0.83	0.71	0.70	0.78

Note. Correlations of 0.14 or less are significant at * *p* < 0.05, while correlations of 0.23 or greater are significant at ** *p* < 0.01.

**Table 2:** Regression Analyses for the Mediation Model Examining Personal Mistakes as a Mediator Between Parental Academic Expectations and Depressive Symptoms.

		Unstandardized Coefficients		Standardized Coefficients			95% Confidence Interval for B	
Model	Predictor	B	SE	*β*	t	*p*	Lower Bound	Upper Bound
1	(Constant)	2.54	0.16	—	16.02	<0.001	2.23	2.85
	PE → PS	0.36	0.04	0.44	8.26	<0.001	0.28	0.45
2	(Constant)	4.67	0.51	—	9.10	<0.001	3.66	5.68
	PE → Dep	−0.06	0.11	−0.04	−0.55	0.58	−0.29	0.16
	PS → Dep	−0.49	0.14	−0.23	−3.53	<0.001	−0.77	−0.22

Note: PE—Parental Academic Expectations; PS—Personal Standards; Dep—Depressive Symptoms.

**Table 3:** Regression Analyses for the Mediation Model Examining Concern Over Mistakes as a Mediator Between Parental Academic Expectations and Depressive Symptoms.

		Unstandardized Coefficients		Standardized Coefficients			95% Confidence Interval for B	
Model	Predictor	B	SE	*β*	t	*p*	Lower Bound	Upper Bound
1	(Constant)	2.25	0.18	—	12.37	<0.001	1.89	2.60
	PE → CM	0.20	0.05	0.23	4.04	<0.001	0.10	0.30
2	(Constant)	2.03	0.45	—	4.52	<0.001	1.15	2.29
	PE → Dep	−0.37	0.10	−0.21	−3.56	<0.001	−0.57	−0.16
	CM → Dep	0.62	0.12	0.30	5.21	<0.001	0.38	0.85

Note: PE—Parental Academic Expectations; CM—Concern Over Mistakes; Dep—Depressive Symptoms.

## Data Availability

The data presented in this study are available on request from the corresponding author due to ethical reasons.
